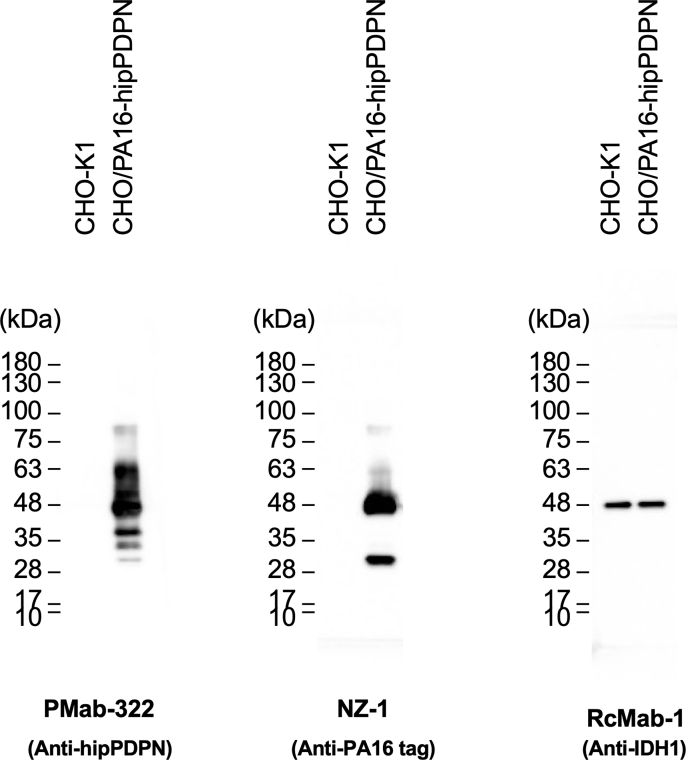# PMab-322: a novel anti-hippopotamus podoplanin monoclonal antibody for multiple applications

**DOI:** 10.1016/j.bbrep.2025.102170

**Published:** 2025-07-23

**Authors:** Haruto Yamamoto, Hiroyuki Suzuki, Tomohiro Tanaka, Mika K. Kaneko, Yukinari Kato

**Affiliations:** Department of Antibody Drug Development, Tohoku University Graduate School of Medicine, 2-1 Seiryo-machi, Aoba-ku, Sendai, Miyagi, 980-8575, Japan

**Keywords:** Hippopotamus podoplanin, Monoclonal antibody, CBIS method, Flow cytometry, Immunoblotting, Immunohistochemistry

## Abstract

Podoplanin (PDPN) is a highly glycosylated type I transmembrane protein. PDPN expression is observed in various normal tissues, including lymphatic endothelial cells, kidney podocytes, and type I alveolar epithelial cells in the lungs. Monoclonal antibodies (mAbs) targeting PDPN across different animal species have facilitated the identification of PDPN-positive cells. To date, we have developed anti-PDPN mAbs for over 20 species. These antibodies suit various applications, including flow cytometry, immunoblotting, and immunohistochemistry. In this study, we generated an anti-hippopotamus PDPN (hipPDPN) mAb, PMab-322 (mouse IgG_2a_, kappa), using the Cell-Based Immunization and Screening (CBIS) method. PMab-322 exhibited strong reactivity to hipPDPN-overexpressed Chinese hamster ovary-K1 and demonstrated moderate affinity (*K*_D_: 4.4 × 10^−8^ M) in a flow cytometry-based measurement. PMab-322 specifically recognizes hipPDPN but does not cross-react with PDPN from 23 other species. Furthermore, PMab-322 successfully detected hipPDPN in both immunoblotting and immunohistochemistry. These findings highlight the potential of PMab-322 for pathological analyses of hippopotamus-derived tissues.

## Highlights

• A novel anti-hippopotamus podoplanin (hipPDPN) monoclonal antibody, PMab-322 was developed by the Cell-Based Immunization and Screening method.
• PMab-322 detects hipPDPN by flow cytometry.
• PMab-322 is also suitable for detecting hipPDPN in immunoblotting and immunohistochemistry.
• PMab-322 could contribute to the pathological analysis of hippopotamus tissues.

## Abbreviations

PDPN: podoplanin
mAb: monoclonal antibody
hipPDPN: hippopotamus podoplanin
CBIS: Cell-Based Immunization and Screening
CHO–K1: Chinese hamster ovary-K1
FBS: fetal bovine serum
BSA: bovine serum albumin
PBS: phosphate-buffered saline

## 1 Introduction

Podoplanin (PDPN) (also known as Aggrus, T1α, E11 antigen, and PA2.26 antigen) is a type I transmembrane protein that has a highly glycosylated extracellular domain, a transmembrane domain, and a short intracellular domain [1,2]. PDPN is expressed in various normal tissues and cells, including lymphatic endothelial cells [3,4], kidney podocytes [5], skin epidermis [6], and lung type I alveolar epithelial cells [7,8].

PDPN is expressed on the apical membrane of lung type I alveolar epithelial cells [7,8]. These cells cover over 95% of the alveolar surface and play a crucial role in gas exchange. During lung development, PDPN expression transitions from being widespread in the embryonic lung epithelium to being specifically localized in type I alveolar cells of the distal epithelium [9]. PDPN-knockout mice showed the lethal phenotype after birth due to respiratory failure. Their lungs fail to properly inflate with air, displaying increased cellular density in the distal lung, abnormal terminal respiratory units, and only a few attenuated type I cells [10,11]. These findings suggest that PDPN is essential for the proliferation and differentiation of lung type I alveolar epithelial cells.

In mammalian skin, PDPN is expressed in lymphatic endothelial cells, the outer root sheath cells of hair follicle keratinocytes, and the basal cell layer of sebaceous glands, but it is absent in the interfollicular epidermis [12]. The keratinocyte-specific PDPN deletion (K5-Cre; PDPN^flox/flox^) in mice exhibited a thicker hair bulb during the mid-anagen to catagen phase, suggesting that the PDPN depletion promotes anagen hair growth [13]. Additionally, hair follicle stem cells isolated from these mice showed reduced focal adhesion and weaker interactions with the extracellular matrix than wild-type mice. This indicates that PDPN loss enhances the migration of hair follicle stem cells toward the bulb area, further supporting its role in stimulating anagen hair growth [13].

There are two species of hippopotamuses: the larger, commonly known as the common hippopotamus (*Hippopotamus amphibius*), and its smaller counterpart, the pygmy hippopotamus (*Choeropsis liberiensis*). They are well-adapted to semi-aquatic environments, enabling them to move efficiently both in water and on land [14]. Molecular data and morphological analyses support the exclusive clade grouping hippopotamuses with cetaceans (whales, dolphins, and porpoises) [[14], [15], [16], [17], [18]]. However, the evolutionary pathway from the hippo-cetacean common ancestor to modern hippopotamuses remains unclear due to the lack of fossil evidence.

The Cell-Based Immunization and Screening (CBIS) method contains the immunization of target antigen-overexpressed cells and high-throughput screening using flow cytometry. Using the CBIS method, various monoclonal antibodies (mAbs) that recognize structural epitope [19], linear epitope [20], and glycosylated epitope [21] of membrane protein have been established. Anti-PDPN mAbs against more than 20 species have been established mainly by the CBIS method (http://www.med-tohoku-antibody.com/topics/001_paper_antibody_PDIS.htm#PDPN). These mAbs contribute not only to the research of each animal, such as a SARS-CoV-2 study [22], but also to diagnosis [23] and drug development [24]. This study aimed to develop anti-hippopotamus PDPN (hipPDPN) mAbs using the CBIS method.

## 2 Materials and methods

### 2.1 Plasmids and cell lines

Synthesized DNA encoding hipPDPN (XM_057709258.1, Eurofins Genomics KK, Tokyo, Japan) plus an N-terminal PA16 tag (GLEGGVAMPGAEDDVV) [25] and an N-terminal MAP16 tag (PGTGDGMVPPGIEDKI) [26], which are recognized by an anti-PA16 tag mAb (NZ-1) [27] and an anti-MAP16 tag mAb (PMab-1) [28], were subcloned into a pCAG-Ble vector [FUJIFILM Wako Pure Chemical Corporation (Wako), Osaka, Japan].

P3X63Ag8U.1 (P3U1) and Chinese hamster ovary (CHO)–K1 cell lines were purchased from the American Type Culture Collection (ATCC, Manassas, VA, USA) and were maintained in Roswell Park Memorial Institute (RPMI)-1640 medium (Nacalai Tesque, Inc., Kyoto, Japan) containing 10% heat-inactivated fetal bovine serum (FBS; Thermo Fisher Scientific, Inc., Waltham, MA, USA), 100 U/mL penicillin, 100 μg/mL streptomycin, and 0.25 μg/mL amphotericin B (Nacalai Tesque, Inc.).

The plasmids were transfected into CHO–K1 as described previously [29]. Stable transfectants (CHO/MAP16-hipPDPN and CHO/PA16-hipPDPN) were maintained in a medium containing 0.5 mg/mL of Zeocin (InvivoGen, San Diego, CA, USA). The information of twenty-four species-PDPN expressed CHO–K1, and the clones of anti-PDPN mAbs are available at the WEB site “Antibody bank” (http://www.med-tohoku-antibody.com/topics/001_paper_antibody_PDIS.htm#PDPN).

### 2.2 Production of hybridomas

The female BALB/cAJcl mice were purchased from CLEA Japan (Tokyo, Japan). Animal experiments were approved by the Animal Care and Use Committee of Tohoku University (Permit number: 2022MdA-001) and were carried out following the NIH (National Research Council) Guide for the Care and Use of Laboratory Animals. Mice were immunized intraperitoneally with 1 × 10^8^ cells/mouse of CHO/MAP16-hipPDPN with Alhydrogel adjuvant 2% (InvivoGen). After four times additional injections, the hybridoma production was performed as described previously [30]. The hybridoma supernatants were screened by flow cytometric analysis using CHO/PA16-hipPDPN and CHO–K1. The hybridoma supernatant containing anti-hipPDPN mAbs in serum-free medium was filtered and purified using Ab-Capcher Extra (ProteNova, Kagawa, Japan).

### 2.3 Flow cytometric analysis

Cells were collected after a brief treatment with 0.25% trypsin and 1 mM EDTA. Afterward, they were rinsed with a blocking buffer of 0.1% bovine serum albumin (BSA) in PBS and incubated with primary mAbs for 30 min at 4°C. Subsequently, the cells were exposed to Alexa Fluor 488-conjugated anti-mouse IgG or anti-rat IgG (1:2,000, Cell Signaling Technology, Inc., Danvers, MA, USA). Fluorescence measurements were then obtained using the SA3800 Cell Analyzer (Sony Corp., Tokyo, Japan).

### 2.4 Determination of apparent dissociation constant (K_D_) by flow cytometry

CHO/PA16-hipPDPN was incubated in a series of diluted PMab-322 solutions for 30 min at 4°C. Then, the cells were treated with Alexa Fluor 488-conjugated anti-mouse IgG at a dilution of 1:200. Fluorescence measurements were obtained using the SA3800 Cell Analyzer. The apparent *K*_D_ was determined by fitting the binding isotherms into the built-in; one-site binding model in GraphPad PRISM 6 software (GraphPad Software, Inc., La Jolla, CA, USA).

### 2.5 Immunoblotting

Immunoblotting was conducted as described previously [30] using 1 μg/mL of PMab-322, 1 μg/mL of NZ-1, or 1 μg/mL of an anti-isocitrate dehydrogenase 1 (IDH1) mAb (RcMab-1) [31] as primary mAbs.

### 2.6 Immunohistochemical analysis

CHO/PA16-hipPDPN and CHO–K1 cell blocks were made using iPGell (Genostaff Co., Ltd., Tokyo, Japan). The paraffin-embedded cell sections were stained with PMab-322 (0.1 μg/mL) using BenchMark ULTRA PLUS with the ultraView Universal DAB Detection Kit (Roche Diagnostics*,* Indianapolis, IN, USA*).*

## 3 Results

### 3.1 Development of anti-hipPDPN mAbs using the CBIS method

**Fig. 1:**
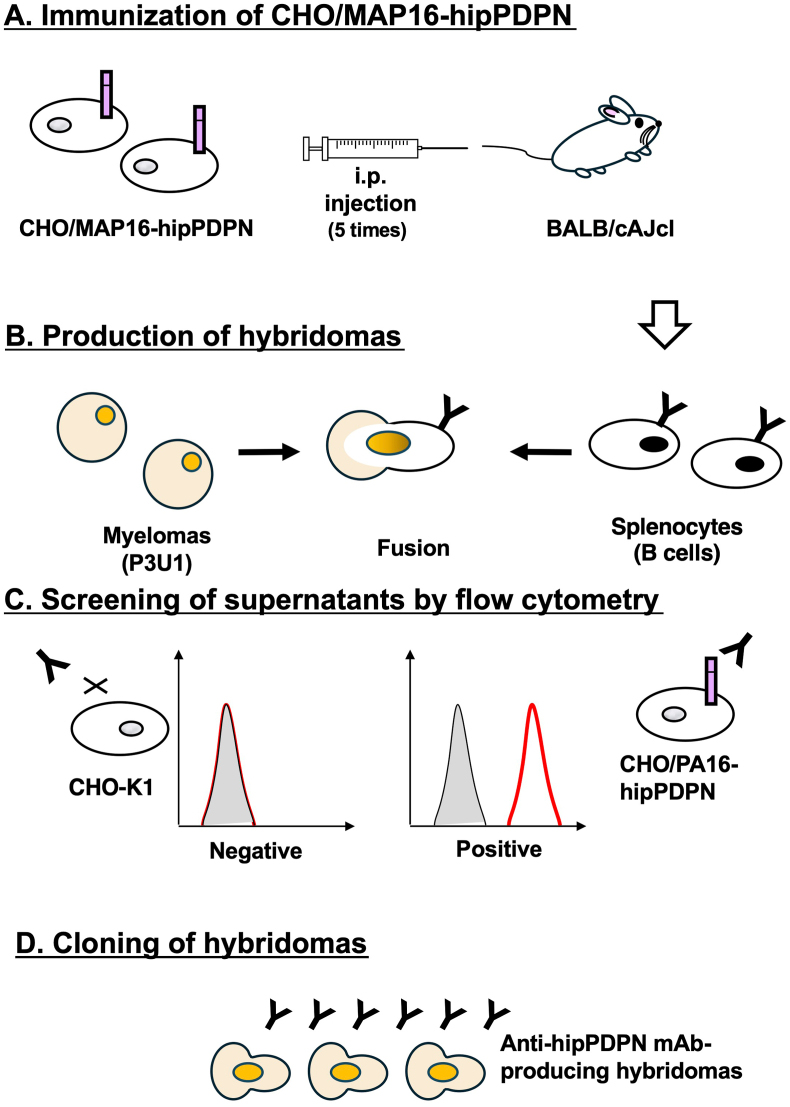
**A schematic illustration of anti-hipPDPN mAbs production.** (**A**) CHO/MAP16-hipPDPN was immunized into BALB/cAJcl mice. (**B**) The spleen cells were fused with P3U1 cells. (**C**) To select anti-hipPDPN mAb-producing hybridomas, the supernatants were screened by flow cytometry using CHO/PA16-hipPDPN and CHO–K1. (**D**) After limiting dilution, anti-hipPDPN mAbs were cloned by limiting dilution. PMab-322 (mouse IgG_2a_, kappa) was finally established.

To produce anti-hipPDPN mAbs, two mice were immunized with CHO/MAP16-hipPDPN (Fig. 1A). After immunization, their spleens were harvested, and the splenocytes were fused with myeloma P3U1 cells (Fig. 1B). The hybridomas were then seeded into ten 96-well plates and cultured for six days. Subsequently, supernatants that exhibited reactivity with CHO/PA16-hipPDPN but not with CHO–K1 were identified from 958 wells using flow cytometry (Fig. 1C). Following limiting dilution and multiple screening steps, a mAb clone, PMab-322 (mouse IgG_2a_, kappa), was successfully generated (Fig. 1D).

### 3.2 Flow cytometry using PMab-322

**Fig. 2:**
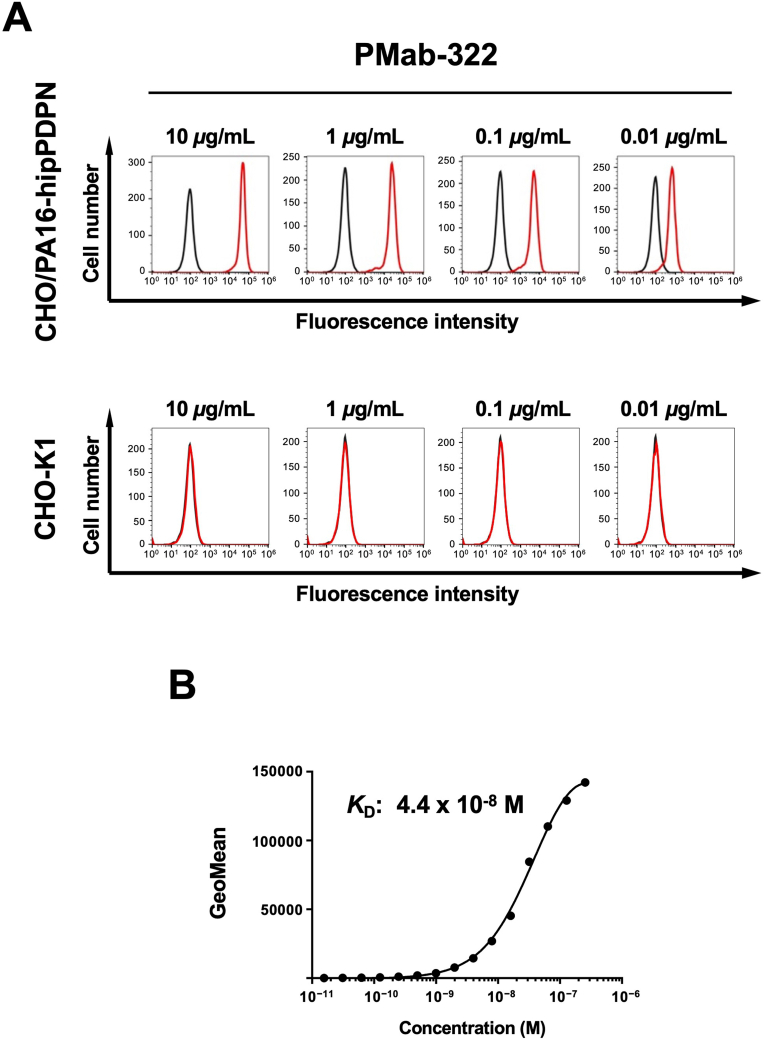
**Flow cytometric analysis of PMab-322 against CHO/PA16-hipPDPN.** (**A**) CHO/PA16-hipPDPN and CHO–K1 were treated with 10–0.01 μg/mL of PMab-322 (red line) or blocking buffer (black line), followed by Alexa Fluor 488-conjugated anti-mouse IgG. (**B**) CHO/PA16-hipPDPN was suspended in 100 μL serially diluted PMab-322. Then, cells were treated with Alexa Fluor 488-conjugated anti-mouse IgG. Fluorescence data were subsequently collected using the SA3800 Cell Analyzer. The dissociation constant (*K*_D_) of PMab-322 was determined by GraphPad PRISM 6.

Flow cytometry was conducted using PMab-322 against CHO/PA16-hipPDPN and parental CHO–K1. PMab-322 showed reactivity to CHO/PA16-hipPDPN (Fig. 2A) from 10 to 0.01 μg/mL. However, PMab-322 did not recognize CHO–K1 even at 10 μg/mL (Fig. 2A). We next conducted flow cytometry to determine the apparent *K*_D_ value of PMab-322 against CHO/PA16-hipPDPN. PMab-322 exhibited a moderate affinity (*K*_D_: 4.4 × 10^−8^ M) to CHO/PA16-hipPDPN (Fig. 2B).

### 3.3 Specificity of PMab-322 against 24 species-PDPN expressed CHO–K1

**Fig. 3:**
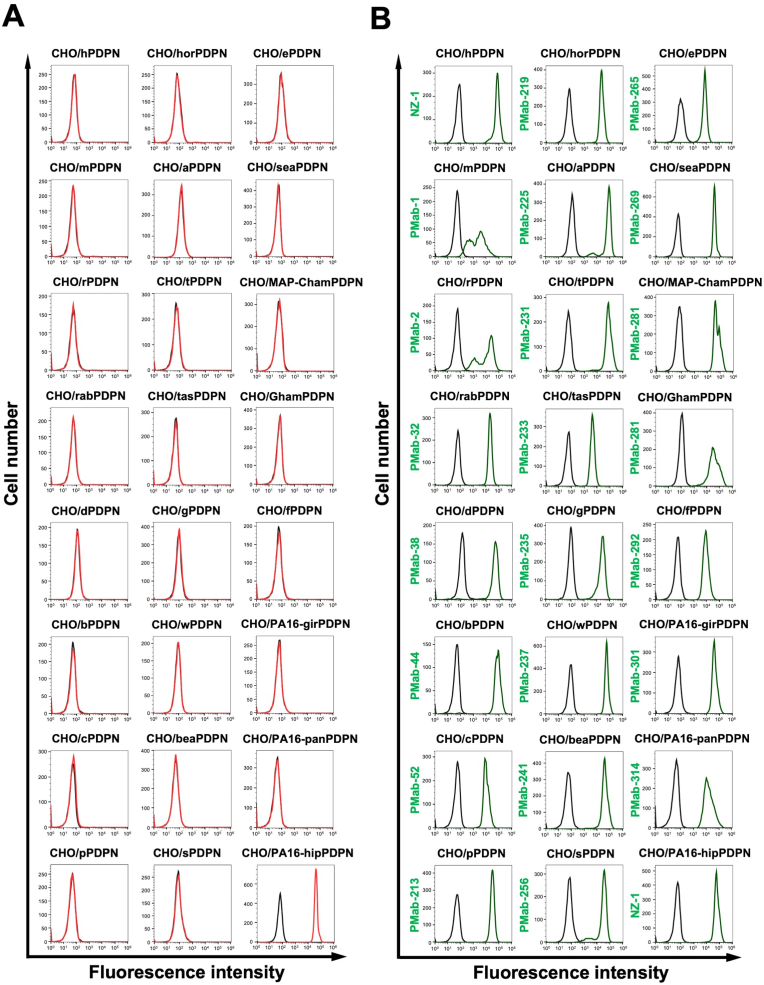
**Specificity of PMab-322 against 24 species PDPN-overexpressed CHO–K1. (A**) Twenty-four species PDPN-overexpressed CHO–K1 cell lines were treated with 10 μg/mL of PMab-322 (red line) or blocking buffer (black line), followed by Alexa Fluor 488-conjugated anti-mouse IgG. **(B**) The expression of each PDPN was confirmed by corresponding anti-PDPN mAbs (green line, 10 μg/mL). Then, Alexa Fluor 488-conjugated anti-mouse IgG or anti-rat IgG were treated. Fluorescence data were collected using the SA3800 Cell Analyzer. Note that the recognition of CHO/PA16-hipPDPN by NZ-1 was mediated by the reaction to PA16-tag.

**Supplementary Fig. S1:**
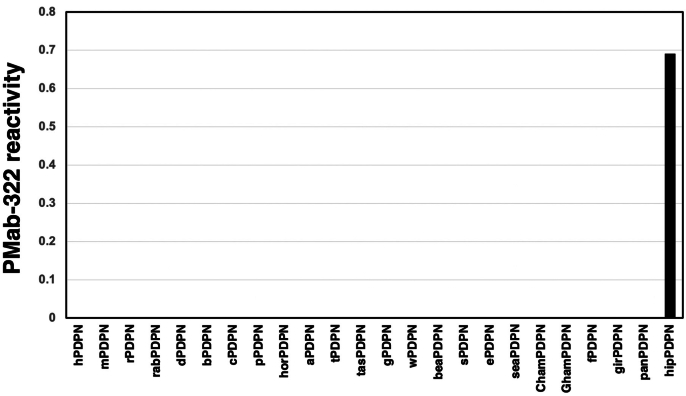
The quantification of the PMab-322 reactivity in CHO–K1 cells, which overexpress PDPN derived from various species. The values (geometric mean of PMab-322 / geometric mean of each control mAb, Fig. 3) were determined.

We previously generated anti-PDPN mAbs against human PDPN (hPDPN, clone NZ-1), mouse PDPN (mPDPN, clone PMab-1), rat PDPN (rPDPN, clone PMab-2), rabbit PDPN (rabPDPN, clone PMab-32), dog PDPN (dPDPN, clone PMab-38), bovine PDPN (bPDPN, clone PMab-44), cat PDPN (cPDPN, clone PMab-52), pig PDPN (pPDPN, clone PMab-213), horse PDPN (horPDPN, clone PMab-219), alpaca PDPN (aPDPN, clone PMab-225), tiger PDPN (tPDPN, clone PMab-231), Tasmanian devil PDPN (tasPDPN, clone PMab-233), goat PDPN (gPDPN, clone PMab-235), whale PDPN (wPDPN, clone PMab-237), bear PDPN (beaPDPN, clone PMab-241), sheep PDPN (sPDPN, clone PMab-256), elephant PDPN (ePDPN, clone PMab-265), sealion PDPN (seaPDPN, clone PMab-269), Chinese hamster PDPN (ChamPDPN, clone PMab-281), Golden hamster PDPN (GhamPDPN, clone PMab-281), ferret PDPN (fPDPN, clone PMab-292), giraffe PDPN (girPDPN, clone PMab-301), panda PDPN (panPDPN, clone PMab-314). We next investigated the reactivity of PMab-322 to each PDPN-overexpressed CHO–K1. As shown in Fig. 3A, PMab-322 solely reacted with hipPDPN, but not others. Cell surface expression was confirmed by mAbs mentioned above (Fig. 3B). The PA16-tagged hipPDPN expression was also confirmed by an anti-PA16 tag mAb, NZ-1 (Fig. 3B). Supplementary Fig. S1 showed the quantification of the reactivity of PMab-322 (geometric mean). These results indicate that PMab-322 specifically recognizes hipPDPN.

### 3.4 Immunoblotting using PMab-322

**Fig. 4:**
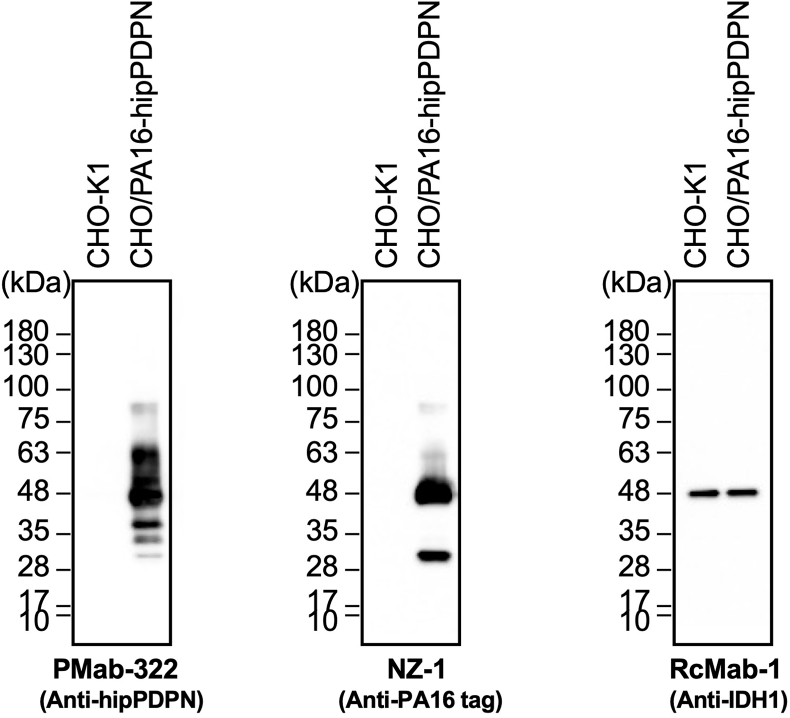
**Detection of hipPDPN by immunoblotting.** The membranes, on which cell lysates of CHO–K1 and CHO/PA16-hipPDPN were transferred, were incubated with 1 μg/mL of PMab-322, 1 μg/mL of NZ-1, or 1 μg/mL of RcMab-1. The membranes were incubated with horseradish peroxidase-conjugated anti-mouse (for PMab-322) or horseradish peroxidase-conjugated anti-rat immunoglobulins (for NZ-1 and RcMab-1). Chemiluminescence signals were developed and detected with a Sayaca-Imager.

We investigated whether PMab-322 can be applied for immunoblotting using CHO/PA16-hipPDPN and CHO–K1 cell lysates. As shown in Fig. 4, PMab-322 could detect hipPDPN as the significant bands around 48–63 kDa in CHO/PA16-hipPDPN cell lysates, while no band was detected in CHO–K1. An anti-PA16 tag mAb, NZ-1, could detect PA16-hipPDPN as the main band around 48 kDa in CHO/PA16-hipPDPN cell lysates. An anti-IDH1 mAb (clone RcMab-1) was used for internal control due to the stable expression. These results indicate that PMab-322 can detect hipPDPN in immunoblotting.

### 3.5 Immunohistochemistry using PMab-322

**Fig. 5:**
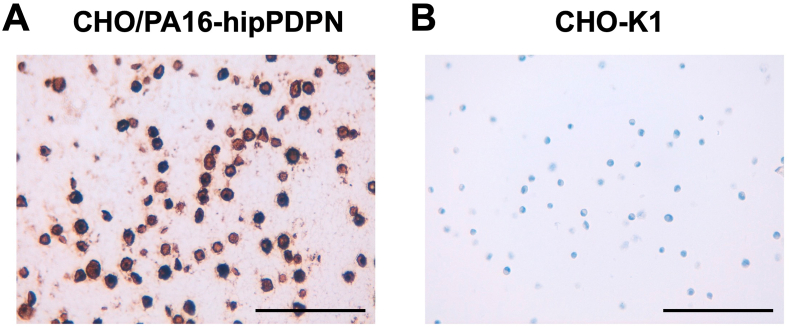
**Immunohistochemistry of paraffin-embedded sections of CHO/PA16-hipPDPN and CHO–K1.** The sections of CHO/PA16-hipPDPN (**A**) and CHO–K1 (**B**) were treated with 0.1 μg/mL of PMab-322. The staining was carried out using BenchMark ULTRA PLUS with the ultraView Universal DAB Detection Kit. Scale bar = 100 μm.

To investigate whether PMab-322 can be used for immunohistochemistry, paraffin-embedded CHO–K1 and CHO/PA16-hipPDPN sections were stained with PMab-322. A membranous staining was observed in CHO/PA16-hipPDPN (Fig. 5A) but not in CHO–K1 (Fig. 5B). These results indicate that PMab-322 is suitable for immunohistochemistry for detecting hipPDPN-positive cells in paraffin-embedded cell samples.

## 4 Discussion

This study first demonstrated a novel and sole anti-hipPDPN mAb, PMab-322 which is highly versatile for basic research. PMab-322 was shown to be useful for flow cytometry and exhibited moderate binding affinity (4.4 × 10^−8^ M) against CHO/PA16-hipPDPN (Fig. 2). PMab-322 specifically recognized hipPDPN but did not react with PDPNs from other species, including whales, the closest living relatives of hippopotamuses (Fig. 3). Furthermore, PMab-322 successfully detected hipPDPN in immunoblotting (Fig. 4) and immunohistochemistry using paraffin-embedded cell blocks (Fig. 5). Therefore, PMab-322 is expected to facilitate the identification of endogenous hipPDPN-positive cells in tissues, such as the kidney, skin, and lung.

In a hamster model of SARS-CoV-2 infection, our developed anti-hamster PDPN mAb, PMab-281, contributed to the pathological analysis to identify the PDPN-positive alveolar differentiation intermediate cells in the affected lung [22]. The SARS-CoV-2 infection in a hippopotamus was reported in 2021. The animal died after onset of clinical signs and SARS-CoV-2 was detected by real-time PCR in the lung, spleen, liver, and intestine samples [32]. PMab-322 would help the pathological analysis of those tissues to understand the pathogenesis of SARS-CoV-2 in hippopotamus.

Mammals have fully adapted to life in the ocean only twice in history: in Cetacea and Sirenia (manatees and dugongs) [15,33]. In the case of Cetacea, genetic studies clearly show that fully aquatic cetaceans and semiaquatic hippopotamus are the closest living relatives [[16], [17], [18]]. Studies suggest that particular water-related adaptations may have evolved in their common ancestor (Cetancodonta: Cetacea + Hippopotamidae) [34]. Still, another possibility is that these traits developed separately in each group [14]. The integumentary systems of cetaceans and hippopotamus were analyzed by integrating comprehensive genomic and histological data. Both cetaceans and hippopotamus have lost function in eight skin-related genes involved in epidermal, hair follicles, and sebaceous glands differentiation [35]. However, none of these genes are shared by cetaceans and hippopotamus. These results support the hypothesis that aquatic skin adaptations evolved independently in cetaceans and hippopotamus [35]. Since PDPN is expressed in skin epidermis and the lymphatic endothelial cells [6], PMab-322 (this study) and PMab-237 (an anti-whale PDPN mAb [29]) would contribute to the molecular and pathological analysis of the skin.

Cetaceans and hippopotamus adapt to the aquatic and semi-aquatic environments, respectively. The lungs of cetaceans have anatomical and physiological adaptations that enable them to hold their breath for extended periods while diving [14]. The immunohistochemical analysis revealed the presence of smooth muscle in the terminal bronchioles, alveolar ducts, and alveolar septa, which is thought to play a key role in the alveolar collapse reflex and prolonged breath-holding during diving [36]. However, the pathological analysis of the lung of hippopotamus has not been reported. PMab-322 and PMab-237 could be valuable tools for comparative histological analyses of the lung by staining lung type I alveolar epithelial cells.

## CRediT authorship contribution statement

**Haruto Yamamoto:** Investigation. **Hiroyuki Suzuki:** Writing – original draft, Investigation. **Tomohiro Tanaka:** Investigation, Funding acquisition. **Mika K. Kaneko:** Conceptualization. **Yukinari Kato:** Project administration, Conceptualization, Writing – review & editing, Funding acquisition.

## Author disclosure statement

The authors have no conflict of interest.

## Funding information

This research was supported in part by 10.13039/100009619Japan Agency for Medical Research and Development (10.13039/100009619AMED) under Grant Numbers: JP25am0521010 (to Y.K.), JP25ama121008 (to Y.K.), JP25ama221339 (to Y.K.), and JP25bm1123027 (to Y.K.), and by the 10.13039/501100001691Japan Society for the Promotion of Science (10.13039/501100001691JSPS) Grants-in-Aid for Scientific Research (10.13039/501100001691KAKENHI) grant nos. 24K18268 (to T.T.) and 25K10553 (to Y.K.).

## Declaration of competing interest

The authors declare that they have no known competing financial interests or personal relationships that could have appeared to influence the work reported in this paper.

## Figures and Tables

**figs2:**